# The Involvement of Insulin-Like Growth Factor 1 and Nerve Growth Factor in Alzheimer’s Disease-Like Pathology and Survival Role of the Mix of Embryonic Proteoglycans: Electrophysiological Fingerprint, Structural Changes and Regulatory Effects on Neurotrophins

**DOI:** 10.3390/ijms22137084

**Published:** 2021-06-30

**Authors:** Michail Aghajanov, Senik Matinyan, Vergine Chavushyan, Margarita Danielyan, Gohar Karapetyan, Margarita Mirumyan, Katarine Fereshetyan, Hayk Harutyunyan, Konstantin Yenkoyan

**Affiliations:** 1 Department of Biochemistry, Yerevan State Medical University after M. Heratsi, Yerevan 0025, Armenia; michail.aghajanov@meduni.am (M.A.); senik.matinyan@gmail.com (S.M.); margaritamirumyan@gmai.com (M.M.); katarinefereshetyan@gmail.com (K.F.); 2 Laboratory of Neuroscience, Yerevan State Medical University after M. Heratsi, Yerevan 0025, Armenia; verginechavushyan@gmail.com (V.C.); margaritadanielyan76@gmail.com (M.D.); googakarapetyan@gmail.com (G.K.); hayk@web.am (H.H.); 3 Laboratory of Neuroendocrine Relations, L. Orbeli Institute of Physiology of NAS, Yerevan 0028, Armenia; 4 Laboratory of Histochemistry and Electromicroscopy, L. Orbeli Institute of Physiology of NAS, Yerevan 0028, Armenia

**Keywords:** amyloid-beta (25–35), IGF-1, NGF, hippocampus, proteoglycans of embryonic genesis (PEG)

## Abstract

Alzheimer’s disease (AD)-associated neurodegeneration is triggered by different fragments of amyloid beta (Aβ). Among them, Aβ (25–35) fragment plays a critical role in the development of neurodegeneration—it reduces synaptic integrity by disruption of excitatory/inhibitory ratio across networks and alters the growth factors synthesis. Thus, in this study, we aimed to identify the involvement of neurotrophic factors—the insulin-like growth factor 1 (IGF-1) and nerve growth factor (NGF)—of AD-like neurodegeneration induced by Aβ (25–35). Taking into account our previous findings on the neuroprotective effects of the mix of proteoglycans of embryonic genesis (PEG), it was suggested to test its regulatory effect on IGF-1 and NGF levels. To evaluate the progress of neurodegeneration, in vivo electrophysiological investigation of synaptic activity disruption of the entorhinal cortex–hippocampus circuit at AD was performed and the potential recovery effects of PEG with relative structural changes were provided. To reveal the direct effects of PEG on brain functional activity, the electrophysiological pattern of the single cells from nucleus supraopticus, sensomotor cortex and hippocampus after acute injection of PEG was examined. Our results demonstrated that after *i.c.v.* injection of Aβ (25–35), the level of NGF decreased in cerebral cortex and hypothalamus, and, in contrast, increased in hippocampus, prompting its multidirectional role in case of brain damage. The concentration of IGF-1 significantly increased in all investigated brain structures. The administration of PEG balanced the growth factor levels accompanied by substantial restoration of neural tissue architecture and synaptic activity. Acute injection of PEG activated the hypothalamic nucleus supraopticus and hippocampal neurons. IGF-1 and NGF levels were found to be elevated in animals receiving PEG in an absence of amyloid exposure. We suggest that IGF-1 and NGF play a critical role in the development of AD. At the same time, it becomes clear that the neuroprotective effects of PEG are likely mediated via the regulation of neurotrophins.

## 1. Introduction

Alzheimer’s disease covers the vast majority of age-related neurodegenerative diseases. Being predominantly, if not exclusively, described with characteristic plaque deposition (mainly consisting of aggregated beta-amyloid), synaptic dysfunction, intracellular neurofibrillary tangles, diffuse loss of neurons and dystrophic neurites, this condition poses a severe threat to the aging population [1]. The inability to convincingly associate this phenomenon with one particular pathogenetic element has led to the emergence of various heavily debated theories.

Accumulated evidence suggests the role of neurotrophins in the neuronal development, network maintenance, plasticity and possible dysregulation in AD-like pathologies. The neurotrophin family member nerve growth factor (NGF) deprivation resulted in Aβ accumulation/deposition, tau hyperphosphorylation, and synaptic dysfunction in mice [2]. Another research has shown β-amyloid precursor protein (APP) as a crucial player in regulating retrograde transport of NGF [3]. In the same study, downregulation of APP lead to reduced cell surface levels of NGF-specific receptors. Selective degeneration of the nucleus basalis cholinergic cortical projection neurons has been associated with dysregulation of NGF high (Tropomyosin receptor kinase A) and low (Neurotrophin receptor P75) affinity receptors [4]. Going forward, experimental evidence established crucial role of Insulin-like growth factors (IGF-1 and IGF-II) in neurogenesis and neurodevelopment [5]. These trophic factors, being especially distributed in the hippocampus, exert neuroprotective properties against amyloid-induced neurotoxicity [6]. A reduced response to insulin signaling has been suggested to be an early feature of AD [7]. This relatively new wave of enthusiasm is being fueled by reports demonstrating reductions in both insulin and IGF-I mRNA expression, and downregulation of the corresponding receptors [1]. The opposing relationship between serum IGF-I and brain Aβ levels questioned the use of IGF-1 as a treatment for AD [8].

For the last decade, our laboratory has been studying the potential neuroprotective ability of a mix of proteoglycans of embryonic genesis (PEG). We have previously shown that administration of PEG leads to the improvement of spatial memory and neurotransmission in amyloid-induced models of AD [9], regulates oxidative stress and the monoamine levels in brainstem, cerebral cortex and hippocampus [10,11]. These findings prompt the hypothesis of PEG as a promising neuroprotective compound.

In our current study, we estimated the IGF-1 and NGF concentrations in an AD-like animal model, their shifts after injection of the mix of proteoglycans of embryonic genesis (PEG) and relative morphological changes. The electrophysiological pattern of the single cells from nucleus supraopticus, sensomotor cortex and hippocampus after acute injection of PEG and the spike train pattern of the hippocampal neurons were provided in a group after amyloid exposure, and under the influence of PEG.

## 2. Results

### 2.1. Biochemical Results

#### 2.1.1. Changes of IGF-1 in Cerebral Cortex, Hippocampus and Hypothalamus

**Figure 1:**
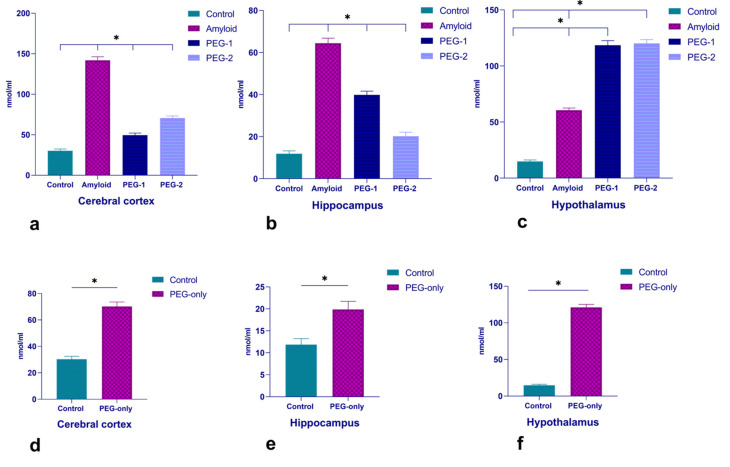
Changes of the IGF-1 concentration at neurodegeneration and under PEG influence. The concentrations of the IGF-1 in control, amyloid, PEG-1 and PEG-2 groups (**a**–**c**), as well as under the influence of PEG only (**d**–**f**). The data are represented in nmol/mL form. *p*-values below 0.05 (*) were considered as statistically significant.

The concentration of IGF-1 significantly elevated in the cerebral cortex, hippocampus and hypothalamus after amyloid exposure (Figure 1). The shift was more pronounced in the cerebral cortex and the hippocampus (Figure 1a,b). The administration of proteoglycans of embryonal genesis effectively dropped down the IGF-1 in the cerebral cortex and the hippocampus. The concentration of IGF-1 increased after PEG injection in the hypothalamus in comparison with the amyloid group (Figure 1c). Proteoglycans of embryonic genesis significantly increased the level of IGF-1 in all the mentioned structures and especially in the hypothalamus, when administered in an absence of amyloid exposure (PEG-only group) (Figure 1d–f).

#### 2.1.2. Changes of NGF in Cerebral Cortex, Hippocampus and Hypothalamus

**Figure 2:**
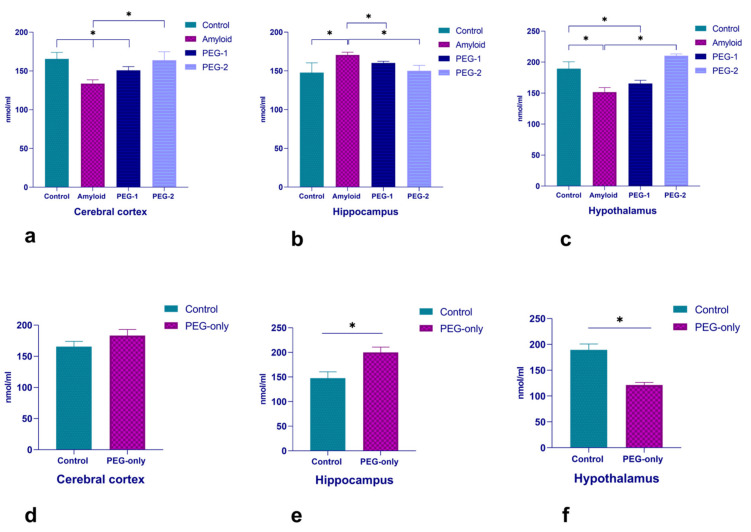
Changes in the NGF concentration with neurodegeneration and under the influence of PEG. The concentrations of the NGF in control, amyloid, PEG-1 and PEG-2 groups (**a**–**c**), as well as under the influence of PEG (**d**–**f**). The data are represented in nmol/mL form. *p*-values below 0.05 (*)were considered as statistically significant.

The NGF concentration has been found to be elevated after amyloid 25–35 injection in the hippocampus and dropped down in the cerebral cortex and hypothalamus (Figure 2a–c). The PEG injection stabilized the initial changes, although the effect was more pronounced after injection of embryonal proteoglycans in PEG-2 mode (Figure 2a–c). The control values were found to be significantly elevated in the PEG-only group, except in the hypothalamus (Figure 2d–f).

### 2.2. Morphological Findings

**Figure 3:**
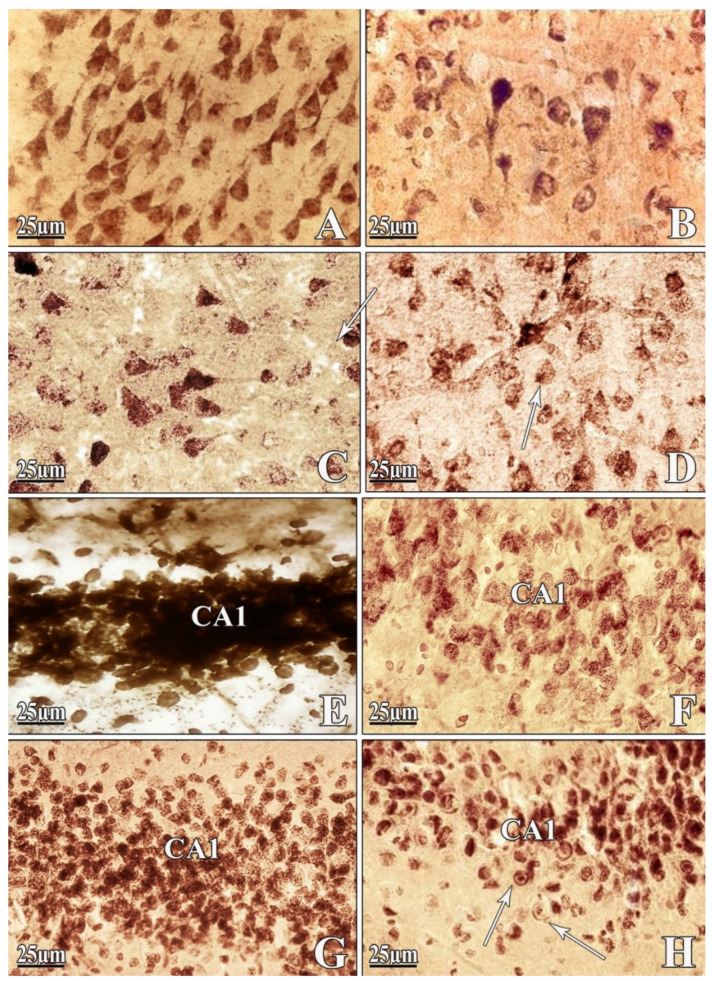
Frontal slices of the cerebral cortex and CA1 region of hippocampus. Pyramidal neurons of the cerebral cortex in normal conditions (**A**), after bilateral *i.c.v.* injection of Aβ 25–35 (**B**), and under the influence of PEG in PEG-1 (**C**) and PEG-2 (**D**) groups. Slices of CA1 neurons of intact rats’ brain hippocampus (**E**), after bilateral *i.c.v.* injection of Aβ 25–35 (**F**) and of PEG-1 (**G**) and PEG-2 groups (**H**). (**B**)—chromatolysis, the eccentrically located nucleus and nucleolus of pyramidal neurons of the cortex; (**C**,**D**)—densely distributed neurons; granulation in soma and processes of neurons of the cortex; (**G**)—fine-grained sediment in soma of dentate gyrus neurons; satellites; (**G**,**H**)—proliferation of neurons in the hippocampal CA1 field (white arrows indicate centrally located nucleus). Magnification: ×400 (**E**–**H**); ×1000 (**A**–**D**).

Some cortical areas after Aβ (25–35) exposure remain relatively intact, and anterior cingulate neurons of the prefrontal cortex are affected more intensively. The granular sediment gradually disappears from the cytoplasm of the most swollen neurons (Figure 3B). As a rule, the nucleus passes to the pole of the cell where sediments are present, so it has a peripheral position (Figure 3B).

Neurons with a major apical dendrite are identified in the pyramidal layer of the cerebral cortex after PEG injection in PEG 1 group (Figure 3C). A large granular sediment and high phosphatase activity are detected in soma and processes, especially in the apical dendritic zone. Some cell nuclei are hyperphosphorylated. The contours of neighboring cells appeared among these neurons (Figure 3C). An increase in the neurons’ density is observed in the PEG-2 group. The form is restored in pyramidal cells, a fine granular sediment appears in the soma and the apical dendrites are directed perpendicular to the cortical surface (Figure 3D).

A similar pattern is observed in the hippocampal sections. In the DG, the processes of neuronal cleavage are accompanied by a spongy alveolar state of cytoplasm. The precipitate disappears in one of the poles of the cell, and the other pole looks intensely colored after amyloid exposure (Figure 3F). The nucleus shows its abnormal state and adaptation to metabolic changes by passing to the periphery, where the sediment grains are still preserved.

After injection of PEG both in preventive and treatment modes, there is pronounced cell proliferation in hippocampal CA1 region, as well as a tendency to restore the shape and size of neurons in the dentate gyrus of the hippocampus, where fine-granular sediment appears in the soma (Figure 3G,H).

**Figure 4:**
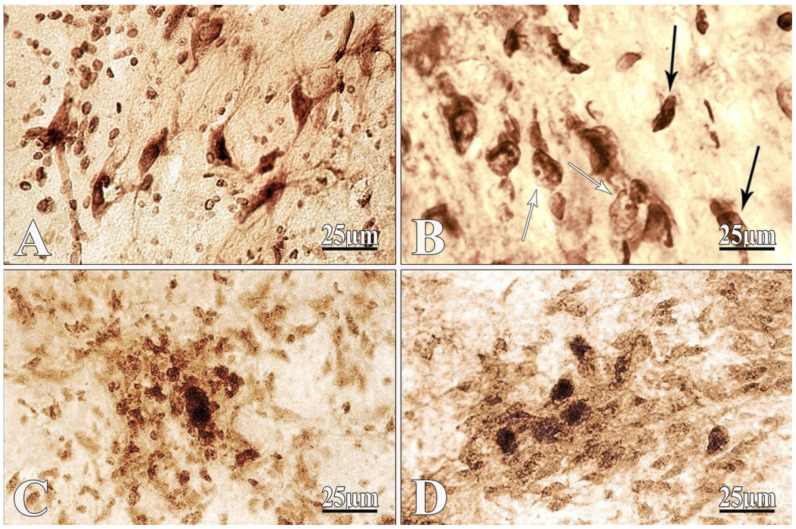
Frontal slices of nucleus basalis of Meynert. Neurons in normal conditions (**A**), after bilateral i.c.v. injection Aβ 25–35 (**B**), in PEG-1 (**C**) and PEG-2 (**D**) groups. (**A**) Triangular and polygonal cells with a centrally located nucleus, with the reaction of the glial cell nuclei; (**B**) the disorder of nerve cells’ shape and size (black arrow) and the eccentrically disposed nucleus of neurons (white arrow); (**C**) dark cluster, surrounded by glia; (**D**) survived neurons. Identification of Ca^2+^-dependent acid phosphatase activity. Magnification: × 400 (**A**–**D**).

Small nerve cells with triangular and polygonal shape with processes were detected on the frontal slices of the nucleus basalis of Meynert in the brain of intact rats; the nuclei occupy a central location and the presence of reactive glial elements is observed (Figure 4A).

The results of the morphological study of rat brain with the intracerebroventricular administration of amyloid peptide Aβ 25–35 revealed a rarefaction in the density of the arrangement of nerve cells in the nucleus basalis of Meynert: the shape of the neurons is disturbed and the processes are not detected (Figure 4B). Nerve cells have black, angular or rod-like formations. The processes are thin and shortened. The apical and lateral dendrites coil or disappear. Clumps of tigroid substance reach each other, merge into a compact, dark-colored mass and the nucleus stretches out (Figure 4B).

In the nucleus, basalis of the Meynert accumulation of glial cell nuclei was observed after PEG injection. Dark, formless clusters were also found, surrounded by glial cells nuclei (Figure 4C). Survived and partially restored neurons were found in the basal nucleus of Meynert after PEG injection in PEG 2 group (Figure 4D).

### 2.3. Electrophysiology

#### 2.3.1. Dynamic Variety/Changes of Spike Activity of Single Neurons of Hippocampus, Nucleus Supraopticus and Sensomotor Cortex Following Acute Injection of PEG

**Figure 5:**
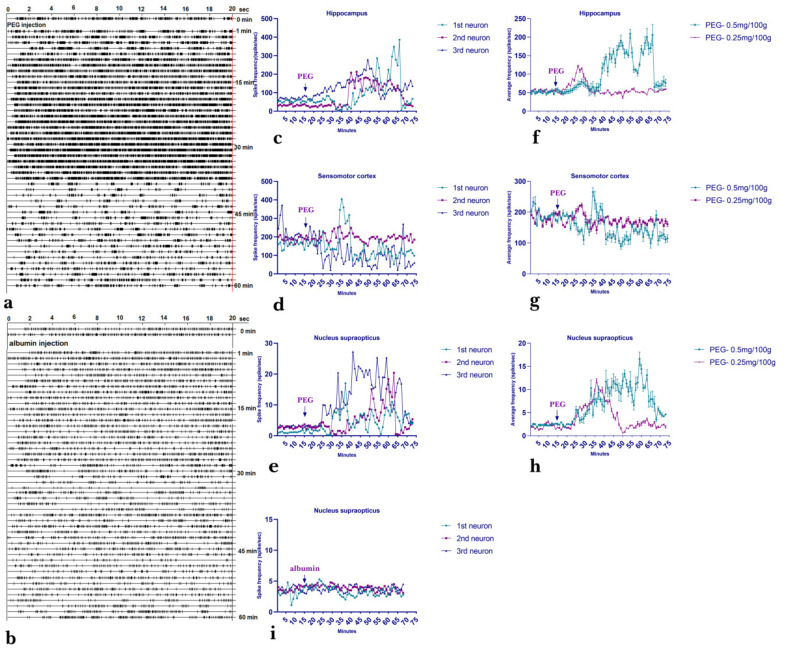
Spike activity of single neurons following acute injection of PEG. Impulse flow of a single hippocampal neuron (shown by the amplitude discriminator selection) in case of PEG (**a**) and albumin (**b**) injection; spike activity is presented as a “raster” in real time (time bin 20 sec). (**c**–**e**) Spike activity changes of single neurons of hippocampus (*n* = 3), sensomotor cortex (*n* = 3) and nucleus supraopticus (*n* = 3), from the initial level to 60 min after i/p injection of PEG (0.5 mg/kg). Abscissa—exposure time of PEG (minute), Ordinate—number of spikes (spike/sec) during registration (time bin 20 sec). Excitation effects are more expressed in hippocampus and nucleus supraopticus. (**f**–**h**) average spike frequency (mean ± SEM) of hippocampal neurons (*n* = 60) and neurons from the sensomotor cortex (*n* = 60) and nucleus supraopticus (*n* = 60) in case of different doses of PEG. (**i**)—spike activity changes of single neurons of nucleus supraopticus (*n* = 3) after albumin injection.

To elucidate the mechanism of PEG action, simultaneous recordings from the hippocampus, supraoptic nucleus (*n.so.*) of the hypothalamus and the sensomotor cortex after acute injection of PEG were performed (Figure 5). For this purpose, PEG was injected intraperitoneally at a dose of 0.5 and 0.25 mg per 100 g of animal weight. The average spike frequency (mean ± SEM) of hippocampal neurons (*n* = 60) and neurons from the sensomotor cortex (*n* = 60) and nucleus supraopticus (*n* = 60) in case of different doses of PEG administration revealed a dose of 0.5 mg per 100 g of body to be more effective (Figure 5f–h). The real-time dynamics in hippocampal neurons of intact animal revealed an increase in spike activity from an average of 15 min and achieving a baseline level at 60 min after injection of a single therapeutic dose of PEG (0.5 mg/100g body weight, i.p.), as well as transformation of the rhythm of background spike activity (Figure 5c).

The most pronounced effect was detected in the recordings from the *n*.so. of the hypothalamus, where the background spike activity increased three times after PEG injection (Figure 5e,h). The spike activity histograms in three single neurons of the hippocampus (*n* = 3 neuron), nucleus supraopticus (*n* = 3 neuron) and sesomotor cortex (*n* = 3 neuron) are demonstrated in Figure 5c–e. The injection of albumin did not change the spike train pattern significantly (Figure 5i).

Thus, we assume that PEG activates the neurosecretory functions of the brain, in particular by influencing the supraoptic nucleus of the hypothalamus.

#### 2.3.2. Excitatory and Inhibitory Responses of Hippocampal Neurons to HFS of Entorhinal Cortex

Overall, 147 hippocampal neurons in the control group, 90 hippocampal neurons in the amyloid group, 96 hippocampal neurons in the PEG 1-treated group and 75 hippocampal neurons in the PEG-2-treated group were recorded. The analysis of spike activity revealed the acceleration of the spike train during HFS (tetanic potentiation, TP) and post-stimulus period (post-tetanic potentiation, PTP), as well as the deceleration of the spike train during HFS (tetanic depression, TD) and post-stimulus period (post-tetanic depression, PTD). The different combinations of responses, such as TP-PTP, TP-PTD, TD-PTD and TD-PTP, were recorded. In each experimental group, the relative contribution of excitatory or inhibitory responses was calculated as a percentage share of the analyzed neurons with the corresponding type of response. The statistical significance was estimated according to the chi square Fisher’s exact test.

**Figure 6:**
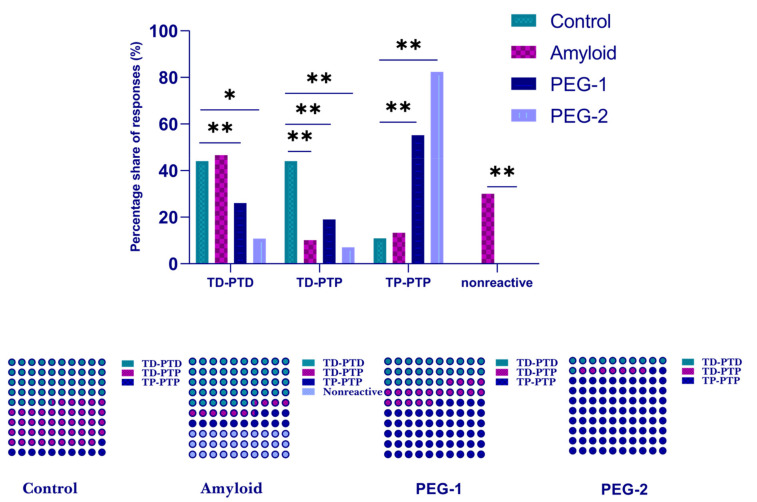
Percentage distribution of responses of hippocampal neurons to HFS of EC at neurodegeneration and under the influence of PEG. Percentage distribution for specified types of responses (TD-PTD, TP-PTP, TD-PTP and nonreactive) in experimental groups control (*n* = 6 rats), amyloid (*n* = 5 rats), PEG-1 (*n* = 5 rats) and PEG-2 (*n* = 5 rats). The statistical significance was estimated according to the chi square test. (* *p* < 0.05, ** *p* < 0.01).

The relative distribution of responses of hippocampal neurons to HFS of EC in amyloid and control groups revealed statistically significant increase in nonreactive neurons (30% or 27 neurons out of 90 vs. 0% or not detected) and decrease in TD-PTP response type (10% vs. 44%, or 9 neurons out of 90 vs. 65 neurons out of 147). At the same time, the differences in TD-PTD (44.6% vs. 44.9%, or 42 neurons out of 90 vs. 66 neurons out of 147) and TP-PTP responses (13.3% vs. 10.9%, or 12 neurons out of 90 vs. 16 neurons out of 147) between amyloid and control groups were found to be not significant (Figure 6). In the PEG 1 group, the distribution of hippocampal neuron responses was as follows: TP-PTP response pattern increased (55.2% vs. 10.9%, or 53 neurons out of 96 vs. 16 neurons out of 147), whereas TD-PTD (26% vs. 44.9%, or 25 neurons out of 96 vs. 66 neurons out of 147) response types decreased. All of these changes were found to be statistically significant. Interestingly, nonreactive neurons were absent in the PEG 1 group (Figure 6). In the PEG 2 group, TP-PTP responses significantly increased up to 82.4% (61 neurons out of 75) compared to 10.9% in control, whereas TD-PTD and TD-PTP responses were decreased down to 10.8% (5 neurons out of 75) and 7% (9 neurons out of 75), respectively (Figure 6). All of these changes were found to be statistically significant.

## 3. Discussion

The dramatic increase of the IGF-1 after amyloid exposure is probably a “cry to survive” compensatory response to brain damage, as an attempt to preserve neurons that are still intact and/or have the potential for survival. At the same time, activation of the IGF-1 mediates the apoptotic cascade, leading to the death of the damaged cells. From a philosophical point of view, both of these mechanisms can be considered as an altruistic approach [12]. Notably, the concentration of IGF-1 changed in brain structures in the following manner: hippocampus > cortex > hypothalamus. The increase of IGF-1 was more expressed in hippocampus, suggesting that the extent of IGF-1 alteration is dependent on severity of damage and the neural population.

In turn, an increase of IGF-1 may indicate its role as one of the main physiological regulators involved in the clearance of cerebral β-amyloid [13]. Interestingly, it has been shown that IGF-I immunoreactivity was seen in a subpopulation of GFAP-immunopositive astroglia in the temporal cortex of post-mortem AD brain, strengthening its neuroprotective role in case of AD [14]. In our study, proteoglycans of embryonic genesis, in an absence of amyloid exposure (PEG-only group) significantly increased the level of IGF-1 (Figure 1). The administration of PEG according to the PEG 1 scheme significantly suppressed the increase of IGF-1 in the cortex and hippocampus. On the other hand, the IGF-1 concentration in the hypothalamus remained at a fairly high level. In fact, the changes of the IGF-1 in the PEG-2 group were of the same character as after the preliminary administration of PEG. Consequently, the mechanism of action of embryonic proteoglycans, regardless of the single or double injection, was suggested as follows: PEG → IGF-1 (hypothalamic) → paracrine regulation of cortical and hippocampal IGF-1, or directly triggered: PEG → IGF-1 (hypothalamic); PEG → IGF-1 (hippocampal); PEG → IGF-1 (cortical). Regardless of the possible mechanism of PEG, it acts as a modulator of IGF-1 in the aforementioned structures. It is also worth noting that the injection of PEG in both variants had a tendency to regulate the level of IGF-1, bringing it to the level registered in the PEG-only group (Figure 1).

Although reduced in the cerebral cortex and the hypothalamus, the NGF-1 was found to be elevated in the hippocampal structures. In our opinion, such bidirectional shifts indicate a non-stereotyped neuronal response to damage. An increase of NGF in the hippocampus during amyloid-induced neurodegeneration can also be considered as a case of long-term neuronal plasticity, when the survival of neurons is achieved via the mobilization of the brain stem cell reserves. The following chain may be triggered in this case: amyloid (neurodegeneration) → NGF ↑ (compensatory) → neurogenesis → neuronal plasticity = survival.

The administration of PEG in both variants brought the altered NGF to the control value, regardless of the direction of NGF initial shift. Naturally, the changes after the double injection of PEG were more pronounced. A non-stereotyped change in the PEG-only group, in all likelihood, is a case of heterogeneity of neural populations involved in response generation.

The activity of nucleus supraopticus increased up to three times in comparison with the initial level after acute injection of PEG. The reason for this could be the triggering effect of PEG to the neurosecretory unit of hypothalamus, which is a matter for future investigations. It is possible that our data, with similar effects and timing manifestation, could be attributed to universality of signal transduction by GPCRs (G protein-coupled receptors). As a chemokine, PEG, through GPCR, may activate the cAMP-dependent pathway, and consequently, we recorded fast transient activation effects in single neurons of hippocampus, nucleus supraopticus and sensomotor cortex.

The increased levels of endogenous Aβ enhances the initial release probability at the CA3-CA1 synapses of the hippocampus, which, in turn, destructs the vesicles and affects the synaptic short-term plasticity and the firing probability of the CA1 output neuronal synapse [15,16]. In our previous study, we have already shown the disruption of short-term plasticity in nucleus basalis magnocellularis (NBM) hippocampus and NBM-basolateral amygdala cholinergic circuits [17]. In contrast to the deficits seen in cholinergic and glutamatergic systems, the non-significant change of TD-PTD response pattern after amyloid exposure can be due to the fact that GABAergic neurons and receptors appear more resistant to neurodegeneration during AD [18]. A significant increase was of the share of nonreactive neurons was present in the amyloid group (30% vs. not detected in control), presumably through a mechanism that involves the endocytosis of AMPA receptors and the subsequent collapse of dendritic spines [19]. It seems reasonable to consider that in order to preserve hippocampal function, surviving hippocampal neurons begin to increase synthesis of GABAA receptor subunits so as to maintain inhibitory hippocampal circuitry.

Our data about the elevation of excitation responses in PEG groups may be explained by antagonistic signaling of synaptic and extrasynaptic NMDARs. Cell death in neurodegenerative diseases may be partly due to an imbalance of synaptic and extrasynaptic NMDAR signaling caused by synapse loss, failure to transduce Ca^2+^ signals from the synapse to the nucleus or by redistributions of NMDARs from synaptic to extrasynaptic sites [20]. The process seems to be regulated after PEG injection, which should be studied in future investigations.

The described morphological state of nerve cells is characterized by the reactive events from the glial elements. In response to amyloid, as under the influence of PEG1, the neuroglia react to pathological changes occurring in the nervous parenchyma in the form of reactive proliferative processes. Neuroglia are very sensitive to changes occurring in the neural tissue; however, in this case, they do not reach regressive states with degenerative glial cleavage.

PEG injection increases the tendency to restore the shape and size of neurons; however, there is a manifestation of the protective reaction of gliocytes in relation to neurons, which corresponds to modern ideas about the existence of a close interaction between neurons and gliocytes as an integral unit.

## 4. Methods

### 4.1. Animals

The experiments were performed on Sprague-Dawley male rats, weighing 220–300 g. They were kept under standard conditions of laboratory vivarium and provided with food ad libitum. The experimental protocol corresponded to the conditions of the European Communities Council Directive (86/609/EEC) and was approved by the Ethics committee of Yerevan State Medical University after Mkhitar Heratsi (15 November 2018, IRB APPROVAL N4).

### 4.2. Experimental Protocol

Animals were divided into five groups (10 animals in each group). The control group consisted of vehicle (double distilled water)-treated animals. The first experimental group was intracerebroventricularly (*i.c.v.*) treated with the 3 µg/100 g body weight aggregated Aβ (25–35) (AD-like group). The animals from the second experimental group (PEG-only) were subcutaneously injected with PEG (0.5 mg/100g body weight). The animals from the third experimental group (PEG-1) were subcutaneously injected with PEG (0.5 mg/100g body weight) 7 days prior to the *i.c.v.* Aβ (25–35) injection. The animals from the fourth experimental group (PEG-2) were subcutaneously injected with PEG (0.5 mg/100g body weight) seven days prior to the *i.c.v.* Aβ (25–35) and on the 30th day after amyloid injection. The rats were sacrificed after intraperitoneal (i.p.) administration of 40 mg/kg of Nembutal. The control, first, third and fourth experimental groups were sacrificed at the 90th day of the experiment; the second experimental group was terminated on the 7th day after PEG injection.

### 4.3. Composition of PEG

PEG consists of the following proteoglycans of embryonic genesis: alpha-fetoprotein, chorionic gonadotrophin, b1-glycoprotein, carci-noembryonic antigen and carbohydrate antigens Ca-19-9 and Ca-125 [9].

### 4.4. Preparation of Aβ (25–35) Peptide

Aβ (25–35) peptide was purchased from Sigma–Aldrich (St. Louis, MO, USA) and aggregated according to the manufacturer’s recommendations [21].

### 4.5. Surgical Procedure for Aβ (25–35) Injection

Surgical procedures were done under Nembutal anesthesia (40 mg/kg). The rats were placed in a stereotaxic apparatus and their skin was shaved and disinfected. The midline incision was performed to expose the scalp. The holes for the injection were determined by coordinates according to the Paxinos and Watson stereotaxic atlas [22].

Animals were injected with 3 µL of sterile double distilled water (vehicle-treated) or 3 µL of aggregated Aβ (25–35) solution into each cerebral lateral ventricle at a rate of 1 µL/min using the peristaltic pump. 

### 4.6. Electrophysiology Studies

#### 4.6.1. Electrophysiological Recordings after Acute Injection of PEG

Once the rats were anesthetized with Urethan (1.2 g/kg) and immobilized with 1% ditiline (25 mg/kg i/p), they were placed in a stereotaxic frame. The animals were connected to the catheter of mechanical ventilation. The glass recording microelectrode (1–2 μm tip diameter) filled with 2 M NaCl was repeatedly inserted into the nucleus supraopticus, sensomotor cortex and hippocampus. The electrophysiological activity was recorded after injection of a single therapeutic dose of PEG (1 mg/kg, 1 mg/mL, intraperitoneally) in a one-hour period. The sham-operated animals (*n* = 6) received albumin (1 mg/kg, 1 mg/mL, intraperitoneally) under the same experimental conditions.

#### 4.6.2. Excitatory and Inhibitory Responses of Hippocampal Neurons to High Frequency Stimulation of Entorhinal Cortex

The rats were injected intraperitoneally with a combination of Urethan (1.2 g/kg) and dilitine (25 mg/kg). Furthermore, they were fixed in a stereotaxic apparatus and connected to the catheter of mechanical ventilation. The stimulating electrode was placed into the ipsilateral entorhinal cortex (EC) according to the following coordinates (AP-9, L±9, DV+4.0 mm). Single cell spike activity was recorded by 1–2 µm tip diameter electrode filled with 2 M NaCl, which was repeatedly placed into dorsal hippocampus according to the coordinates (AP-3.3, L±1.5–3.5, DV+3.0–4.0 mm). High frequency stimulation (HFC) (100 Hz during 1s) was performed by means of rectangle pulses of 0.05 ms duration and 0.08–0.16 mA amplitude. Automated program was used for mathematical analysis of recorded spikes, which were sorted by amplitude discrimination and all artifacts were excluded.

### 4.7. Biochemical Studies

#### 4.7.1. Sample Preparation

The brain structures were dissected according to the stereotaxic atlas of Paxinos and Watson [22], then frozen in liquid nitrogen, weighed and homogenized in a specific buffer containing 1000 mM Tris-HCl, 2% serum albumin, 1M NaCl, 4 mM EDTANa2, 2% Triton (TM) X-100, 0.1% sodium azid and protease inhibitors.

#### 4.7.2. Assessment of IGF-1 and NGF Using the Enzyme Linked Immunosorbent Assay

For the assessment of IGF-1 and NGF, commercially available kits for ELISA have been used, i.e., Diagnostic Systems Laboratories, Inc., Webster, TX, USA (Active Mouse/Rat IGF–I EIA, DSL-10-2900) and ChemiKineTM, USA (Nerve Growth Factor, Sandwich ELISA Kit, Cat. No. CYT 304), respectively. The assessment and graphical representation were performed on Multiskan System (Thermo Fischer Scientific, Greenville, NC, USA) according to the manual.

### 4.8. Morphological Study

Perfusion-fixed brain samples were collected for further morphological study. To identify and visualize the structural features of single neurons, a modified method of Nissl staining and Golgi silver impregnation was used [23].

For the fixation and consequent preparation of the brain slices, we have used a previously adopted protocol, described in [23].

### 4.9. Statistical Analysis

We used Shapiro–Wilk test to check the normal distribution of the data; moreover, a t-test, one-way ANOVA and Tukey post hoc tests were applied for comparative studies. To increase the reliability in electrophysiological studies of statistical evaluations, we also used the non-parametric method of verification by application of the Wilcoxon two-sample test, taking into account the asymptotic normality of this criterion and allowing a comparison of the calculated values with the table values of the standard normal distribution. The statistical significance for the individual response types of the hippocampal neurons was estimated according to the chi square test. *p*-values below 0.05 were considered as statistically significant. SPSS software package (v. 23) was used to perform the statistical analyses.

## 5. Conclusions

Our findings clearly prove that IGF-1 and NGF play an important, yet not fully covered role in the development of AD. Their changes can be considered as a mirror of functional manifestation of intracellular changes. PEG regulates the amyloid-induced changes, stabilizes the neural response and triggers subsequent recovery via regulation the level of neurotrophic factors.

## Data Availability

Data can be made available by the corresponding author upon reasonable request.
